# Long-Term Effectiveness of Oral Ferric Maltol vs Intravenous Ferric Carboxymaltose for the Treatment of Iron-Deficiency Anemia in Patients With Inflammatory Bowel Disease: A Randomized Controlled Noninferiority Trial

**DOI:** 10.1093/ibd/izab073

**Published:** 2021-05-14

**Authors:** Stefanie Howaldt, Eugeni Domènech, Nicholas Martinez, Carsten Schmidt, Bernd Bokemeyer

**Affiliations:** 1 Research Institute for IBD–HaFCED e.K., Hamburg, Germany; 2 Gastroenterology and Hepatology Department, Hospital Universitari Germans Trias i Pujol, Badalona, Catalonia, and Centro de Investigación Biomédica en Red en Enfermedades Hepáticas y Digestivas, Madrid, Spain; 3 Gastroenterology Research of America, San Antonio, Texas, USA; 4 Department of Gastroenterology, Hepatology, Endocrinology, Diabetology, and Infectious Diseases, Klinikum Fulda, Fulda, Germany; 5 Gastroenterology Practice Minden and University Hospital Schleswig-Holstein, Campus Kiel, Clinic for Internal Medicine I, Kiel, Germany

**Keywords:** ferric maltol, iron replacement, oral therapy, hemoglobin, tolerability

## Abstract

**Background:**

Iron-deficiency anemia is common in inflammatory bowel disease, requiring oral or intravenous iron replacement therapy. Treatment with standard oral irons is limited by poor absorption and gastrointestinal toxicity. Ferric maltol is an oral iron designed for improved absorption and tolerability.

**Methods:**

In this open-label, phase 3b trial (EudraCT 2015-002496-26 and NCT02680756), adults with nonseverely active inflammatory bowel disease and iron-deficiency anemia (hemoglobin, 8.0-11.0/12.0 g/dL [women/men]; ferritin, <30 ng/mL/<100 ng/mL with transferrin saturation <20%) were randomized to oral ferric maltol 30 mg twice daily or intravenous ferric carboxymaltose given according to each center’s standard practice. The primary endpoint was a hemoglobin responder rate (≥2 g/dL increase or normalization) at week 12, with a 20% noninferiority limit in the intent-to-treat and per-protocol populations.

**Results:**

For the intent-to-treat (ferric maltol, n = 125/ferric carboxymaltose, n = 125) and per-protocol (n = 78/88) analyses, week 12 responder rates were 67% and 68%, respectively, for ferric maltol vs 84% and 85%, respectively, for ferric carboxymaltose. As the confidence intervals crossed the noninferiority margin, the primary endpoint was not met. Mean hemoglobin increases at weeks 12, 24, and 52 were 2.5 vs 3.0 g/dL, 2.9 vs 2.8 g/dL, and 2.7 vs 2.8 g/dL with ferric maltol vs ferric carboxymaltose. Treatment-emergent adverse events occurred in 59% and 36% of patients, respectively, and resulted in treatment discontinuation in 10% and 3% of patients, respectively.

**Conclusions:**

Ferric maltol achieved clinically relevant increases in hemoglobin but did not show noninferiority vs ferric carboxymaltose at week 12. Both treatments had comparable long-term effectiveness for hemoglobin and ferritin over 52 weeks and were well tolerated.

## Graphical Abstract

**Graphical Abstract:**
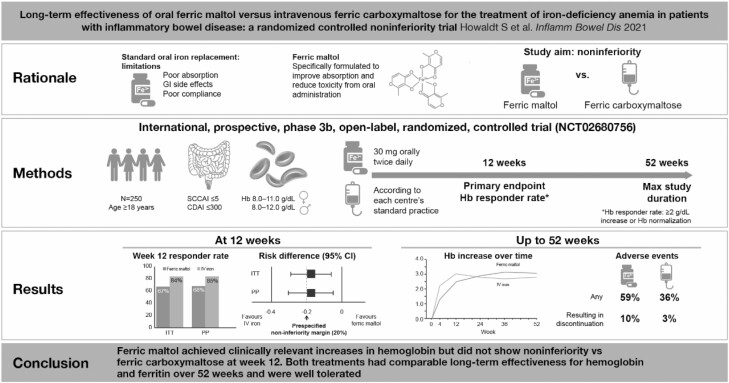

## Introduction

An estimated 36% to 90% of patients with inflammatory bowel disease (IBD) have iron deficiency because of chronic inflammation, mucosal blood loss, and iron malabsorption.^1^ Iron deficiency is associated with fatigue, headache, dizziness, shortness of breath, tachycardia, reduced cognitive function, and depression, with a substantial negative impact on patients’ day-to-day functioning, ability to work, and quality of life.^2^

Treatment of iron-deficiency anemia (IDA) involves iron replacement therapy, typically with oral ferrous iron preparations.^3^ However, the use of these compounds may be limited by poor bioavailability of ferrous salts and gastrointestinal adverse events, particularly in patients with IBD.^3, 4^ After oral administration, up to 90% of ferrous iron is unabsorbed and undergoes oxidation in the gut, resulting in the generation of reactive oxygen species that can cause mucosal damage and gastrointestinal adverse events.^5^ Reflecting this risk, oral ferrous compounds are contraindicated in patients with ulcerative colitis in some countries,^6-8^ and even in patients who are eligible for oral iron replacement therapy, compliance can be poor.^9^

Intravenous (IV) iron is typically used in patients with more severe anemia (hemoglobin [Hb] < 10 g/dL), clinically active IBD, or previous intolerance to oral iron, and in those on erythropoiesis-stimulating agents (ESAs).^10^ Research has shown that IV iron facilitates rapid Hb increases and repletion of body iron stores, even in the presence of inflammation, but it is associated with increased health care costs vs oral iron, along with the inconvenience of clinic visits for infusion, a small but potentially serious risk of anaphylactic reactions, a significantly higher rate of infection vs oral or no iron supplementation, an increase in hypophosphatemia (particularly with ferric carboxymaltose), and a risk of iron overload, because IV iron infusion bypasses the normal physiological mechanisms regulating iron levels.^3, 11, 12^

Ferric maltol is a chemically stable complex of ferric iron and maltol, specifically formulated for improved absorption from oral administration; ferric iron is delivered to the intestinal mucosa in a biologically labile complex, allowing the efficient uptake of elemental ferric iron into enterocytes at a relatively low daily dose while avoiding free iron in the gut, thereby minimizing gastrointestinal toxicity.^13-19^ In phase 3 clinical trials in patients with quiescent or mild to moderate IBD and mild to moderate IDA, ferric maltol provided highly statistically significant and clinically meaningful improvements in Hb vs placebo within 12 weeks^20^ that were maintained for up to 64 weeks.^21^ The rate of gastrointestinal adverse events with ferric maltol was low and similar to that seen with placebo.^20^

Given this favorable benefit/risk profile, ferric maltol may offer an effective iron replacement option in patients in whom oral ferrous irons are unsuitable, who would otherwise require IV iron. The aim of the present noninferiority trial was to compare ferric maltol and IV ferric carboxymaltose for the treatment of IDA in patients with IBD in line with usual practice.

## MATERIALS AND METHODS

### Trial Design

This prospective, phase 3b, open-label, randomized controlled trial (EudraCT 2015-002496-26 and NCT02680756) ran between January 2016 and January 2019 in 56 sites in the United States, Germany, Spain, France, Hungary, and Belgium. The trial conduct complied with Good Clinical Practice guidelines, the Declaration of Helsinki, and all applicable country-specific laws and regulations. The protocol was approved by the relevant independent ethics committees and institutional review boards, and all patients provided informed consent before participation. The full protocol is available at https://clinicaltrials.gov/ProvidedDocs/56/NCT02680756/Prot_000.pdf.

### Patients

Patients aged 18 years or older, with quiescent or mild to moderate IBD, were eligible for inclusion if they had IDA and were considered suitable for IV iron treatment by the investigator. We defined IDA as Hb 8 to 11 g/dL for women or 8 to 12 g/dL for men and either ferritin <30 ng/mL or ferritin <100 ng/mL with transferrin saturation <20%.

Patients were excluded if they had anemia unrelated to iron deficiency or had received intramuscular, IV, or depot iron preparations within 8 weeks, oral iron for anemia within 4 weeks, or blood transfusions within 2 weeks before screening. Additional exclusion criteria were more active IBD (Simple Clinical Colitis Activity Index score >5 or Crohn’s Disease Activity Index score >300 during screening), vitamin B_12_ or folic acid deficiency (unless on replacement therapy starting ≥2 weeks before screening), concomitant medical conditions with significant active bleeding likely to initiate or prolong anemia, severe renal impairment (creatinine clearance <30 mL/min, U.S. sites only), or any medical condition that might compromise the safety of the patient or interfere with compliance. Women could not be pregnant or breastfeeding and had to use a reliable method of contraception during the trial and for 4 weeks after their final visit.

### Treatment

Eligible patients were randomized 1:1 to oral ferric maltol or IV ferric carboxymaltose. Randomization was done centrally and was stratified by screening Hb (<10 or ≥10 g/dL for women and <11 or ≥11 g/dL for men) and IBD subtype (ulcerative colitis or Crohn disease).

Ferric maltol was taken orally at a dose of 30 mg twice daily for ≥12 weeks. Patients were instructed to take capsules with water in the morning ≥1 hour before food or concomitant medications and at night ≥2 hours after food or concomitant medications, with ≥8 hours between doses. Patients started IV ferric carboxymaltose, administered according to local prescribing information, within 5 days of randomization. Patients randomized to ferric carboxymaltose could receive additional IV iron from week 12 if they became anemic. Patients had to be withdrawn from the trial if Hb concentrations fell to ≤7.5 g/dL.

Concomitant ESAs (if the dose was stable for 3 months before randomization), vitamin B_12_ and folic acid replacement, and immunosuppressants (if they did not contribute to anemia or affect erythropoiesis) were permitted during the trial.

### Assessments

Initially, the trial was planned to last 52 weeks, but recruitment was slower than expected, in part because of patients’ concerns about the long-term commitment. Therefore, the protocol was amended during the trial, after approximately 80% of participants had been randomized, to remove long-term efficacy and safety follow-up for any new participants entering the study. The primary endpoint and week 12 secondary endpoints remained the same, and there was no impact on sample size. Participants who had already started the trial before the protocol amendment had their final visit at week 12 or the next scheduled visit if after week 12. Long-term efficacy was evaluated in participants who completed 6 and 12 months.

The primary endpoint was Hb responder rate, defined as the proportion of patients achieving either a ≥2 g/dL Hb increase or Hb normalization (women ≥12 g/dL; men ≥13 g/dL) at week 12. Secondary endpoints included the Hb change from baseline to week 12, the proportion of patients achieving Hb increases of ≥1 g/dL and ≥2 g/dL at week 12, the proportion of patients achieving Hb within normal limits at week 12, the change in ferritin from baseline to week 12, the change in efficacy endpoints at 6 and 12 months, and adverse events. Blood samples for Hb assessments were taken at baseline and weeks 4 and 12 for all patients and at weeks 24, 36, and 52 for those who reached these time points. The Medical Outcomes Study 36-Item Short Form Health Survey (SF-36) was administered at baseline and week 12 for all patients and at weeks 24, 36, and 52 for patients who reached these time points. All patients had an end-of-study/early-discontinuation telephone call 14 days after the last study visit, during which any adverse events were recorded. Clinical laboratory tests were analyzed by a central laboratory.

### Statistical Analysis

The null hypothesis was that the difference in responder rate between the ferric maltol and ferric carboxymaltose groups would be 20% or more. The noninferiority margin of 20% was chosen on the basis of clinical judgment and previous studies of IV iron. Allowing for protocol deviations, a sample size of 121 patients per treatment group was calculated to provide 90% power, assuming that the response rate in the ferric maltol group was 75%. The null hypothesis was rejected if the lower limit of the 2-sided 95% confidence interval (CI) for the risk difference at week 12 was below –20% in the intent-to-treat (ITT) and per-protocol (PP) analyses. The CI was calculated using the delta method based on a logistic regression model, adjusted for treatment group, baseline Hb (below vs at least the observed median), and IBD subgroup (ulcerative colitis or Crohn disease).

Secondary efficacy endpoints were analyzed using an analysis of covariance model to calculate the difference in the treatment group least-squares mean (LSM) and the corresponding 95% CIs and *P* values. In a posthoc analysis, time to first additional IV iron (ie, beyond the first planned IV infusions scheduled according to local prescribing information and standard practice) was assessed using Kaplan-Meier plots for patients enrolled in the 52-week protocol. Patients who did not receive additional IV iron were censored at their study end. The mean total amount of IV iron taken during the trial was summarized descriptively.

The primary efficacy analysis was performed for the ITT and PP populations, as is usual for a noninferiority test. All other analyses are presented for the ITT population. The ITT population included all randomized patients, but analyses excluded efficacy measurements obtained after a patient experienced a serious adverse event of hemorrhage or received blood transfusion, received IV iron outside the protocol dose, or started ESA therapy during the trial. The PP population was predefined to exclude patients with major protocol deviations within the first 12 weeks (see Supplementary Data Content) or those with no week 12 visit or no Hb measurement at week 12. For the ITT population up to week 12, missing values were imputed using multiple imputation rather than last observation carried forward (which could tend to decrease any difference between treatments); the PP analysis used an observed-patients approach. Safety and tolerability were analyzed in the safety population (all patients who received ≥1 dose of the study drug) using descriptive statistics, according to the actual treatment received.

Statistical tests were 2-sided, with a significance level of 0.05. The CIs were calculated at the 95% level, reflecting a type I error rate of 0.05. Statistical analyses were conducted using SAS 9.3 (SAS Institute, Cary, NC).

## RESULTS

### Patient Disposition

**Figure 1.**
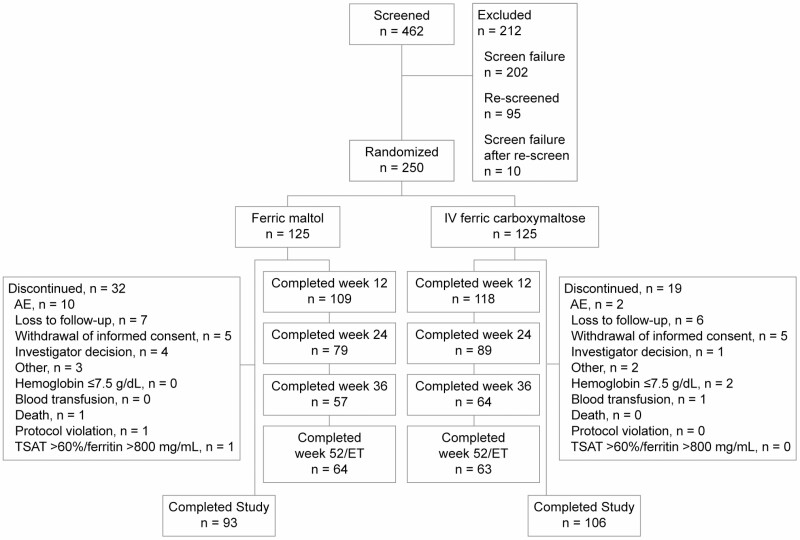
Patient disposition. AE indicates adverse event; ET, end of treatment; TSAT, transferrin saturation.

The ITT population comprised 250 patients, who were randomized to ferric maltol (n = 125) or ferric carboxymaltose (n = 125; Fig. 1). The PP population included 166 patients (ferric maltol, n = 78; ferric carboxymaltose, n = 88), excluding patients with major protocol deviations in the first 12 weeks (see Supplementary Table 1). Two patients randomized to IV iron were not treated (1 was withdrawn after being randomized in error; 1 withdrew consent), and 1 patient randomized to ferric maltol was not treated and was lost to follow-up; in addition, 3 patients were wrongly allocated to treatment (all were randomized to IV iron but erroneously received ferric maltol). Therefore, the safety population comprised 247 patients: 127 in the ferric maltol group and 120 in the ferric carboxymaltose group.

### Baseline Characteristics

Demographic and baseline characteristics are presented in Table 1. The 2 groups were generally similar except for the sex ratio: in the oral ferric maltol group, the prevalence of women was 54% vs 46% male; the ferric carboxymaltose group was composed predominantly of women (62% vs 38%). On average, patients had mildly active IBD, with similar means and ranges of activity scores between treatment groups. Overall, similar proportions of patients received treatment for IBD. At screening, just more than half of the patients (54% in each group) had moderate to severe anemia (Hb < 10 g/dL in women, <11 g/dL in men^22, 23^).

**Table 1.** Demographic and Baseline Characteristics

	Oral Ferric Maltol		IV Ferric Carboxymaltose	
	ITT (n = 125)	PP (n = 78)^*^	ITT (n = 125)	PP (n = 88)^*^
Age, y, mean (SD)	40.0 (14.6)	41.4 (15.3)	40.4 (15.5)	40.2 (15.9)
Range	18-81	18-81	19-77	19-77
Sex, n (%)				
Male	57 (46)	33 (42)	48 (38)	29 (33)
Female	68 (54)	45 (58)	77 (62)	59 (67)
Race, n (%)				
White	110 (88)	72 (92)	111 (89)	78 (89)
Black	6 (5)	2 (3)	3 (2)	3 (3)
Asian	0	0	2 (2)	2 (2)
Other	9 (7)	4 (5)	9 (7)	5 (6)
IBD subgroup, n (%)				
Crohn disease^†^	79 (63)	46 (63)	79 (63)	54 (61)
Ulcerative colitis	46 (37)	29 (37)	46 (37)	34 (39)
IBD activity scores				
CDAI, mean (SD)	129.6 (60.1)	130.9 (60.3)	140.5 (75.8)	130.7 (63.6)
(range)	(0-294)	(37-294)	(33-339)	(36-283)
SCCAI, mean (SD)	2.2 (1.8)	2.0 (1.7)	2.3 (1.6)	2.4 (1.7)
(range)	(0-5)	(0-5)	(0-5)	(0-5)
Hb, g/dL^‡^				
Mean (SD)	10.0 (1.1)	10.0 (1.0)	10.1 (1.0)	10.1 (1.1)
Median (range)	10.1 (7.6-12.6)	10.2 (7.6-12.2)	10.2 (8.0-12.3)	10.1 (8.0-12.3)
Hb <10 g/dL (women) or <11 g/dL (men), n (%)	67 (54)	43 (55)	67 (54)	47 (53)
Ferritin, ng/mL				
Mean (SD)	16.6 (71.6)	9.6 (11.5)	9.3 (12.2)	10.3 (13.8)
Median (range)	6.0 (2.0-797.2)	5.4 (2.0-66.0)	5.8 (2.0-76.0)	6.0 (2.0-76.0)
Concomitant vitamin B_12_ and/or folic acid, n (%)^§^	31 (24)		20 (17)	
Concomitant IBD medications, n (%)^§^				
Corticosteroids				
Systemic	39 (31)		31 (26)	
Topical	3 (2)		6 (5)	
Anti-inflammatory				
Mesalamine	55 (43)		48 (40)	
Immunomodulator				
Azathioprine	32 (25)		37 (31)	
Biologics				
Infliximab	25 (20)		27 (23)	
Adalimumab	28 (22)		19 (16)	
Vedolizumab	18 (14)		13 (11)	

*Patient numbers for the PP populations are the numbers completing 12 weeks of treatment.

^†^Four patients had CDAI scores > 300 (exclusion criterion) and were randomized in error; these patients were included in the ITT analysis but not the PP analysis.

^‡^Three patients had Hb < 8 g/dL at baseline; all 3 patients had Hb ≥ 8 g/dL at screening, thus meeting the eligibility criteria.

^§^Data for concomitant medications are assessed in the safety population: ferric maltol n = 127, ferric carboxymaltose n = 120.

CDAI indicates Crohn’s Disease Activity Index; SCCAI, Simple Clinical Colitis Activity Index.

### Treatment Exposure

In total, 109 patients (87%) in the ferric maltol group and 118 (94%) in the ferric carboxymaltose group completed 12 weeks of treatment; 93 (74%) and 106 (85%), respectively, completed the trial with a scheduled final visit at or after week 12. The most frequent reasons for trial discontinuation were adverse events, loss to follow-up, and withdrawal of informed consent (Fig. 1).

Mean (SD) treatment exposure was 30.2 (17.9) weeks for ferric maltol and 15.5 (15.6) weeks for ferric carboxymaltose, reflecting the different treatment schedules for the study drugs (twice-daily oral therapy vs intermittent IV iron given as required after the initial infusion). The median compliance with ferric maltol was 96.8% at week 12, 96.4% at week 24, 95.8% at week 36, and 98.1% at week 52. Up to week 12, IV iron recipients received between 500 mg and 2500 mg of ferric carboxymaltol (median, 1500 mg; 500-1500 mg per injection) over 1 to 5 injections (median 2 injections), depending on the severity of the anemia and local treatment guidelines. Sixty-eight of 120 patients treated with ferric carboxymaltose received the originally planned IV iron dose, as calculated by the treating physician according to the local prescribing information and standard practice, 21 patients received more than the planned dose, and 31 received less. Up to week 52, 341 IV iron infusions were given to 119 patients. The median total IV iron administered to patients in the ferric carboxymaltose group who completed 52 weeks of treatment (n = 55) was 2000 mg, with a wide range from a minimum of 1000 mg to a maximum of 5500 mg.

### Efficacy

#### Responder rates (ITT and PP populations)

At week 12, in the ITT population, the Hb responder rate (≥2 g/dL increase or normalization in Hb) was 67% in the ferric maltol group and 84% in the ferric carboxymaltose group; the risk difference was –0.17 (95% CI, –0.28 to –0.06; *P* = 0.298). In the PP population, the responder rates were 68% and 85%, respectively; the risk difference was –0.17 (95% CI, –0.30 to 0.05; *P* = 0.341). Because the CIs in both the ITT and the PP analyses crossed the prespecified 20% noninferiority margin, the primary endpoint of noninferiority was not met at week 12.

**Figure 2.**
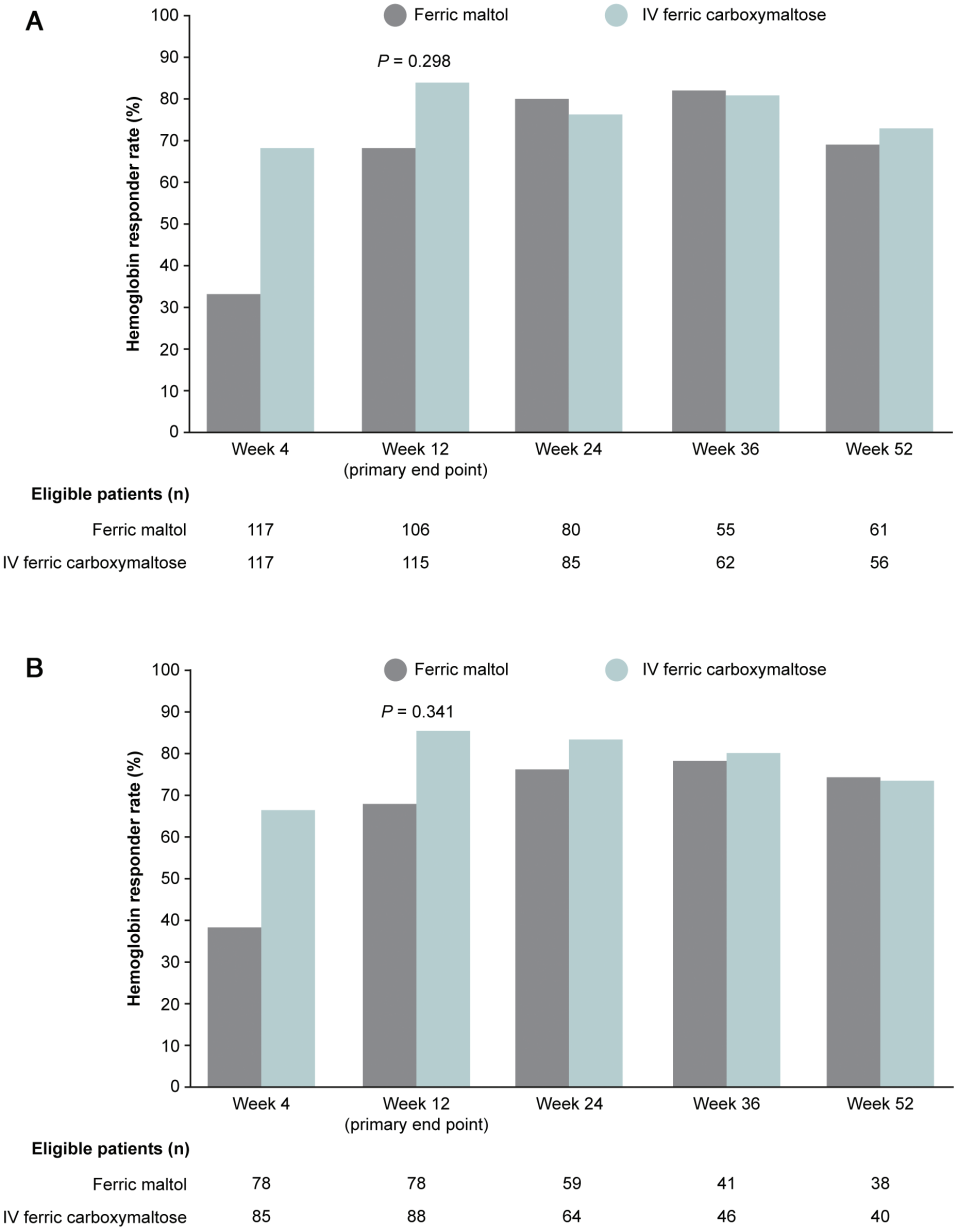
Hb responder rate over 52 weeks of treatment with oral ferric maltol or IV ferric carboxymaltose. (A) ITT population, with multiple imputation for missing values up to week 12. (B) PP population, observed patients. Response was defined as a ≥2 g/dL rise in Hb or normalization of Hb (≥12 g/dL in women, ≥13 g/dL in men). *P* values shown for test of null hypothesis of inferiority in risk difference with noninferiority margin of 20%.

The time profile of the Hb response differed initially between treatments (Fig. 2). At week 4, the responder rate in the ITT population was lower in the ferric maltol group (33%) than in the ferric carboxymaltose group (68%), but the increase in the responder rate from week 4 to week 12 was greater with ferric maltol than with ferric carboxymaltose. From week 24 onward, the responder rates were similar with ferric maltol and ferric carboxymaltose and both treatment groups achieved sustained Hb increases up to week 52 (week 24: 80% vs 76%; week 36: 82% vs 81%; week 52/end of treatment: 69% vs 73% for ferric maltol vs ferric carboxymaltose, respectively).

#### Hb (ITT population)

At baseline, the mean (SD) Hb was 10.0 (1.1) g/dL in the ferric maltol group and 10.1 (1.0) g/dL in the ferric carboxymaltose group. At week 12, the mean (SD) Hb increased to 12.5 (1.6) g/dL in the ferric maltol group and to 13.2 (1.4) g/dL in the ferric carboxymaltose group. The LSM Hb changes from baseline to week 12 were 2.5 and 3.1 g/dL, respectively. The LSM difference between the treatment groups was –0.6 g/dL (95% CI, –1.0 to –0.2; *P* = 0.002), indicating a larger increase in Hb with ferric carboxymaltose than with ferric maltol.

**Figure 3.**
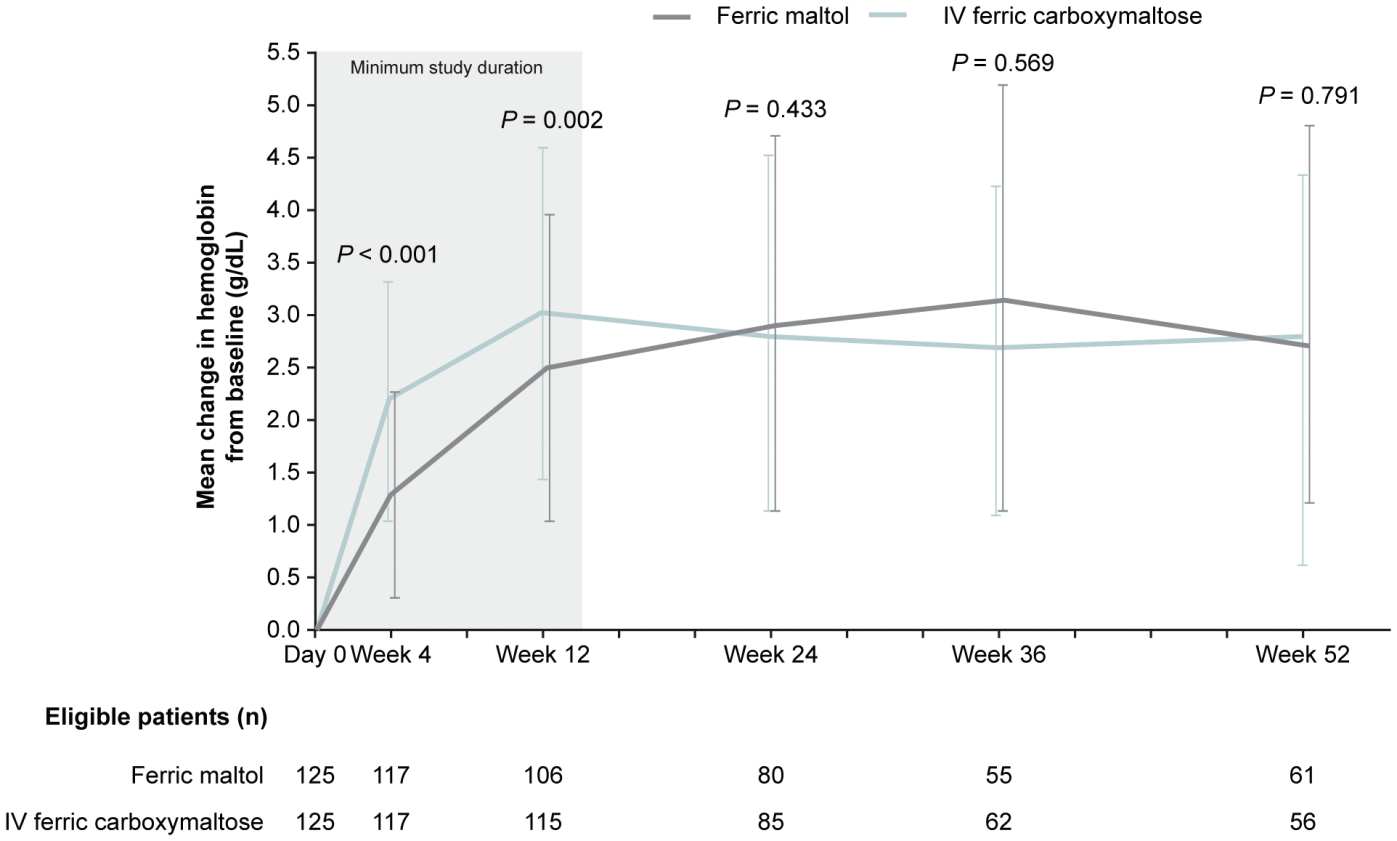
Mean change in Hb over 52 weeks of treatment with oral ferric maltol or IV ferric carboxymaltose (ITT population). *P* values shown for least-squares mean change from baseline, difference between groups.

The initial Hb increase from baseline was faster in the ferric carboxymaltose group than in the ferric maltol group (mean [SD] Hb at week 4, 11.2 [1.4] g/dL with ferric maltol vs 12.3 [1.1] g/dL with ferric carboxymaltose; LSM change from baseline, 1.3 vs 2.2 g/dL; LSM difference between groups, –0.9 [95% CI, –1.2 to –0.7; *P* < 0.001]). By week 24, Hb concentrations converged (mean [SD] Hb at week 24, 12.9 [1.8] g/dL with ferric maltol vs 13.0 [1.6] g/dL with ferric carboxymaltose; LSM change from baseline, 2.7 vs 2.9 g/dL; LSM difference between groups, –0.2 [95% CI, –0.7 to 0.3; *P* = 0.433]). Improvement in Hb was maintained until the end of the study in both groups (mean [SD] Hb at week 52/end of study, 12.8 [2.1] g/dL with ferric maltol vs 13.0 [1.6] g/dL with ferric carboxymaltose; LSM change from baseline, 2.8 g/dL vs 2.9 g/dL; LSM difference between groups, –0.1 [95% CI, –0.8 to 0.6; *P* = 0.791]; Fig. 3).

**Figure 4.**
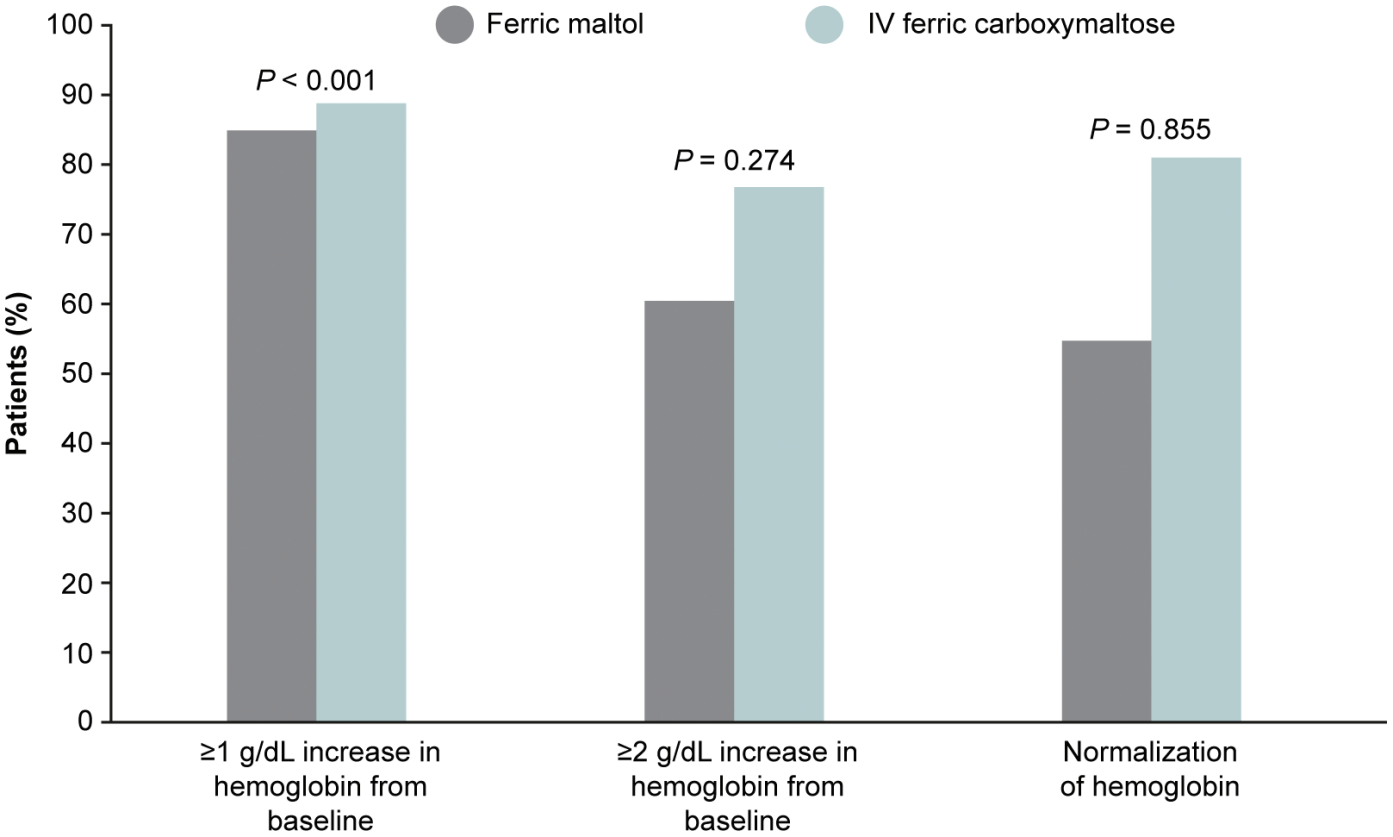
Patients achieving ≥1 and ≥2 g/dL increases, and normalization of Hb concentration between baseline and week 12 (ITT population with multiple imputation). *P* values shown for risk difference between groups.

At week 12, 85% of ferric maltol recipients and 89% of ferric carboxymaltose recipients had a ≥1 g/dL Hb increase from baseline, and 61% and 77%, respectively, had a ≥2 g/dL increase (Fig. 4). The risk difference for the ≥1 g/dL Hb increase was –0.04 (95% CI, –0.12 to 0.05; *P < *0.001), indicating the noninferiority of ferric maltol vs ferric carboxymaltose; the risk difference for the ≥2 g/dL increase was –0.16 (95% CI, –0.28 to –0.04; *P* = 0.274). The Hb was within the normal range at week 12 in 55% of ferric maltol recipients and 81% of ferric carboxymaltose recipients, with a risk difference of –0.26 (95% CI, –0.38 to –0.15; *P* = 0.855; Fig. 4).

#### Iron indices (ITT population)

At baseline, the mean (SD) ferritin concentration was 16.6 (71.6) ng/mL in the ferric maltol group and 9.2 (12.1) ng/mL in the ferric carboxymaltose group. At week 12, the mean (SD) ferritin increased to 25.7 (24.1) ng/mL in the ferric maltol group and to 139.2 (174.6) ng/mL in the ferric carboxymaltose group. The LSM change in ferritin from baseline to week 12 was significantly smaller with ferric maltol than with ferric carboxymaltose (13.7 ng/mL vs 126.7 ng/mL; LSM difference, –113.1 ng/mL; 95% CI, –145.9 to –80.2; *P < *0.001).

As with Hb, ferritin increased steadily with ferric maltol throughout the trial (mean [SD] 19.7 [17.3] ng/mL at week 4, 42.4 [38.4] ng/mL at week 24, 51.8 [53.4] ng/mL at week 36, and 78.9 [141.8] ng/mL at week 52/end of treatment), whereas a sharp increase in ferritin was seen with ferric carboxymaltose at week 4, decreasing thereafter (mean [SD] 158.7 [157.1] ng/mL at week 4, 116.6 [128.9] ng/mL at week 24, 109.9 [159.6] ng/mL at week 36, and 103.4 [143.0] ng/mL at week 52/end of treatment; Supplementary Fig. 1). Normalization of ferritin concentrations was achieved in 46% of ferric maltol recipients vs 81% of ferric carboxymaltose recipients at week 4, 60% vs 76% at week 12, 73% vs 70% at week 24 (*P* < 0.001), 80% vs 60% at week 36 (*P* < 0.001), and 67% vs 70% at week 52 or end of treatment (*P* = 0.025; Supplementary Fig. 2).

#### Additional IV iron (posthoc analysis)

**Figure 5.**
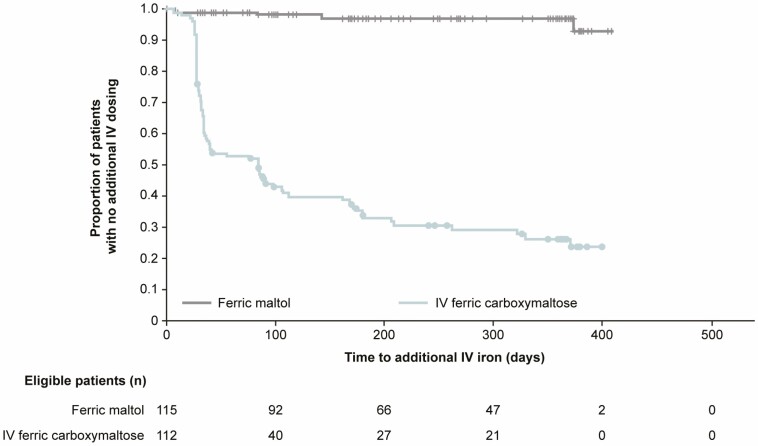
Time to use of additional IV iron after first planned infusions scheduled according to local standard practice and prescribing information (ITT population, posthoc analysis).

After the first planned infusions scheduled according to local prescribing information and standard practice, 85 patients (68%) in the ferric carboxymaltose group had 178 additional IV infusions over the 52 weeks of the trial, with a median time to additional IV iron of 84 days (Fig. 5).

### Tolerability and Adverse Events

Treatment-emergent adverse events (TEAEs; ie, any event starting or worsening on or after the day of the first dose up to 14 days after the last dose) were recorded in 75 patients (59%) receiving ferric maltol and 43 (36%) receiving ferric carboxymaltose, of which 15 were severe (ferric maltol, n = 11; ferric carboxymaltose, n = 4). The TEAEs were mainly gastrointestinal in the ferric maltol group (n = 40 [31%]) and infections/infestations in the ferric carboxymaltose group (n = 22 [18%]; Table 2). Seven patients (5%) on ferric maltol and 8 (4%) on IV iron had an IBD flare; only 1 of these events (in a patient receiving ferric maltol) was deemed to be treatment-related. TEAEs deemed by the investigators to be related to study medication occurred in 32 patients (ferric maltol, n = 25 [20%]; ferric carboxymaltose, n = 7 [6%]). The most frequently recorded treatment-related TEAEs were nausea in 5 patients (ferric maltol, n = 3; ferric carboxymaltose, n = 2) and upper abdominal pain in 5 patients (all on ferric maltol).

**Table 2.** Summary of Adverse Events Occurring or Worsening On or After the First Dose of Study Medication Up to 14 Days After the Last Dose (safety population)

Patients With Adverse Events, n (%)	Ferric Maltol (n = 127)	IV Ferric Carboxymaltose (n = 120)
TEAE	75 (59)	43 (36)
TESAE	12 (9)	4 (3)
Death	1 (<1)	0
Treatment-related TEAE	25 (20)	7 (6)
Treatment-related TESAE	0	0
TEAE leading to discontinuation	13 (10)	3 (3)
TEAEs in ≥2% of patients		
Abdominal pain	12 (9)	3 (3)
Nausea	6 (5)	2 (2)
Abdominal pain upper	7 (6)	2 (2)
Ulcerative colitis flare	4 (3)	4 (3)
Crohn disease flare	3 (2)	4 (3)
Diarrhea	6 (5)	1 (<1)
Constipation	5 (4)	1 (<1)
Feces discolored	4 (3)	0
Flatulence	4 (3)	0
Vomiting	1 (<1)	3 (3)
Nasopharyngitis	10 (8)	4 (3)
Upper respiratory tract infection	1 (<1)	3 (3)
Urinary tract infection	2 (2)	2 (2)
Pyrexia	1 (<1)	4 (3)
Asthenia	3 (2)	1 (<1)
Headache	4 (3)	1 (<1)
Arthralgia	4 (3)	1 (<1)

TESAE indicates treatment-emergent serious adverse event.

Treatment-emergent serious adverse events were reported in 12 patients (9%) receiving ferric maltol and 4 (3%) receiving ferric carboxymaltose; none as deemed related to the study treatment. During the trial, an 82-year-old patient receiving ferric maltol died from natural causes unrelated to the study drug.

Study medication was discontinued prematurely because of adverse events in 13 patients (10%) on ferric maltol (abdominal pain, n = 3; abdominal distension, n = 2; constipation, n = 2; Crohn disease flare, n = 2; and nausea, n = 2) and in 3 patients (3%) on ferric carboxymaltose (hypersensitivity reaction, n = 1; ulcerative colitis flare, n = 1; and adenocarcinoma of colon, n = 1). In addition, 2 patients, both on IV iron, had Hb levels that fell to ≤7.5 g/dL, requiring discontinuation from the trial in accordance with the protocol. There were no clinically meaningful trends in changes in routine clinical laboratory parameters, vital signs, or physical examination findings in either treatment group.

### Quality of Life

According to SF-36 scores, health-related quality of life improved from baseline to week 12 in both treatment groups. Improvements in physical and mental component summary scores were slightly greater with ferric maltol than with IV iron. For physical component summary scores, the mean change from baseline to week 12 was 3.9 with oral ferric maltol and 2.5 with IV ferric carboxymaltose (LSM difference, 1.3; *P* = 0.13). For mental component summary scores, the mean changes were 4.3 and 2.8, respectively (LSM difference, 1.5; *P* = 0.12).

## Discussion

This trial was the first comparative study of oral ferric maltol vs IV ferric carboxymaltose, used according to local prescribing information to reflect real-world practice, in patients with quiescent or mild to moderate IBD and mild to severe IDA. For the primary endpoint of the Hb responder rate at week 12, ferric maltol did not show noninferiority to IV ferric carboxymaltose (ITT and PP analyses) because of the slower time required to achieve an increase in Hb using ferric maltol than using IV iron. Noteworthy is that both groups achieved clinically meaningful increases in Hb after 12 weeks. Mean Hb increases for ferric maltol vs ferric carboxymaltose were 2.5 g/dL vs 3.0 g/dL. Studies have shown that IV administration can bypass physiological iron uptake mechanisms,^3, 24^ whereas ferric maltol relies on the availability of iron transporters in the gut lumen.^16, 18^ As a result, although iron uptake was initially slower than with IV iron replacement, Hb and iron storage measures were increased and sustained over time up to 52 weeks to clinically meaningful levels that were consistent with IV administration, even in patients with moderate to severe Hb levels (≤8.5 g/dL) at baseline. By contrast, many patients in the ferric carboxymaltose group required several IV infusions to sustain Hb increases, and 2 patients on IV iron had to stop treatment in accordance with the protocol when their Hb concentration dropped below 7.5 g/dL. These findings support the use of IV iron in settings where urgent iron replacement is required, such as surgical interventions, whereas either IV iron or oral ferric maltol may be considered for long-term control of chronic iron deficiency, with iron replacement choice determined by resource availability, tolerability profile, and patient preference.

In patients with IBD, IV iron formulations have been compared with oral ferrous iron in 4 parallel-group, randomized controlled trials.^25-29^ A Bayesian network meta-analysis of these trials showed a significant superiority of ferric carboxymaltose over traditional oral ferrous iron (odds ratio, 1.9; 95% credible interval, 1.2-3.2), whereas other IV formulations did not reach statistical significance.^29^ Only 1 trial used IV ferric carboxymaltose, which was compared with oral ferrous sulfate 100 mg twice daily over 12 weeks.^26^ Improvements in Hb were 3.7 and 2.8 g/dL in patients receiving IV ferric carboxymaltose and oral ferrous sulfate, respectively. However, patients in that trial were considerably more anemic at baseline (median Hb, 8.7 g/dL [range, 5.0-11.5] and 9.1 g/dL [range, 5.3-11.1] for ferric carboxymaltose and ferrous sulfate, respectively) than in our trial (median Hb, 10.1 g/dL [range, 7.6-12.6] and 10.2 g/dL [range, 8.0-12.0] for ferric maltol and ferric carboxymaltose, respectively; see Table 1). Data are also available from a 12-week randomized comparison of IV ferric carboxymaltose and IV iron sucrose in patients with mean (SD) baseline Hb concentrations of 10.1 (1.5) g/dL and 10.3 (1.5) g/dL, respectively; 72.8% and 61.8% of patients achieved normalization of Hb.^30^ Improvements in Hb over 12 weeks with ferric carboxymaltose in these studies seem similar in terms of magnitude to those in the current trial. Our study was designed to be consistent with these previous IV ferric carboxymaltose trials, with minor differences reflecting changes in clinical practice over time; our trial was specifically designed to reflect clinical practice by allowing ferric carboxymaltose administration according to local prescribing information. However, caution is advised when comparing results because of differences in trial design, including disease severity at baseline, elemental iron doses, treatment duration, and primary endpoints.

The increase in ferritin concentration at week 12 in the current trial was smaller with ferric maltol than with ferric carboxymaltose. Ferritin concentrations with ferric maltol continued to increase slowly during the course of the trial and were maintained within normal limits for approximately three-quarters of patients during long-term treatment. This pattern is consistent with physiological correction of ferritin concentrations over time. In contrast, ferric carboxymaltose rapidly increased ferritin concentrations by week 4, followed by a decline thereafter. In general, continued exposure to supraphysiological levels of iron carries the risk of iron overload and potential organ damage over time (as suggested by radiographic analyses of liver iron concentrations in patients with end-stage renal disease).^31^ The clinical implications of iron toxicity are still debated and must be considered in the light of blood loss through continuous intestinal bleeding in IBD.^10^ As yet, there are no published radiographic studies of liver iron concentrations in patients with IBD receiving long-term IV iron.^31^

Ferric maltol was generally well tolerated, with only 10% of patients stopping because of adverse events. The frequency of any TEAEs and treatment-related TEAEs was greater with ferric maltol than with ferric carboxymaltose. In particular, gastrointestinal adverse events were more frequent with oral iron than with IV iron, but the rate with ferric maltol was generally consistent with that reported with placebo in previous phase 3 trials of ferric maltol, with the exception of constipation.^20^ When considering the tolerability profiles of the 2 treatments studied, it is important to bear in mind the differences in treatment exposure; ferric maltol was taken daily at a fixed dose, whereas the dose and frequency of IV iron injections varied in the ferric carboxymaltose group, depending on local prescribing practices and patient needs. These aspects should be considered when discussing iron replacement options with patients. Nonetheless, the adverse events reported for patients receiving ferric maltol did not seem to have a deleterious impact on quality of life; ferric maltol was comparable to IV iron in improving SF-36 physical and mental component scores over long-term treatment. Moreover, improvements in the role of physical and general health components were greater in the ferric maltol group than in the IV iron group.

### Limitations

This open-label trial involved treatments with markedly different schedules (daily oral therapy vs periodic injections as needed). As a result, early withdrawals from the ferric maltol arm required extensive imputations for missing data in the ITT analysis, with potential underestimation of the true effect of oral treatment; by contrast, the IV analysis was more likely to be based on actual recorded data, meaning that the comparison was between assumptions and facts. To address this issue, the primary analysis was done in both the ITT and the PP populations. The International Council for Harmonization of Technical Requirements for Pharmaceuticals for Human Use advises caution in the use of ITT analyses in noninferiority trials,^32^ and numerous publications address the benefits and risks of potential alternatives, including PP analyses.^33-37^ However, the PP analysis may have overestimated the effectiveness of ferric maltol in the clinic because it removed patients who withdrew early or had poor compliance. In our analysis, consistent patterns of results between the ITT and PP populations indicated that the findings reflected the true efficacy of the treatments at week 12. Over a longer time course, such as the 52 weeks of this study, the ITT population was likely to be more closely aligned with the real-world experience of treating patients with ferric maltol and ferric carboxymaltose.

The results of the trial may have also been affected by the lack of endoscopic or biomarker assessment of IBD severity during the trial and the higher proportion of women in the ferric carboxymaltose group. Mucosal inflammation could be expected to limit oral iron absorption, and the extent of mucosal inflammation in our patients was unknown; however, the low rate of ulcerative colitis or Crohn disease flare reported as adverse events during the trial indicated that patients had relatively stable disease. The imbalance in the male/female ratio between the treatment groups (women accounted for 54% of the ferric maltol group vs 62% of the ferric carboxymaltose group) was likely to favor ferric carboxymaltose because women have a lower Hb normalization threshold to reach than men (12 vs 13 g/dL), even though both sexes could have started with Hb as low as 8 g/dL, and women tend to respond better to iron replacement therapy than men.^38^ Thus, the ferric maltol group, with proportionally fewer women, would be expected to have lower rates of Hb normalization than the ferric carboxymaltose group, in line with the results of this trial.

## Conclusions

In this first comparative trial reflecting real-world conditions in patients with quiescent or mild to moderate IBD and mild to severe IDA, both oral ferric maltol and standard regimens of IV ferric carboxymaltose achieved clinically meaningful increases in Hb over 12 weeks of treatment, although ferric maltol did not meet the prespecified noninferiority margin vs IV iron. Over the longer term, ferric maltol showed comparable efficacy in maintaining Hb improvements and increasing ferritin up to week 52, consistent with IV iron. The safety profile of each treatment was consistent with previous studies. Thus, ferric maltol offers simple, tolerable, long-term treatment of chronic IDA in patients with IBD.

## Supplementary Material

izab073_suppl_Supplementary_Material

## Abbreviations

CI: confidence interval
ESA: erythropoiesis-stimulating agent
Hb: hemoglobin
IBD: inflammatory bowel disease
IDA: iron-deficiency anemia
ITT: intent-to-treat
IV: intravenous
LSM: least-squares mean
PP: per-protocol
SF-36: Medical Outcomes Study 36-Item Short Form Health Survey
TEAE: treatment-emergent adverse event

## Notes

Author contributions: All authors made substantial contributions to the study design. SH, ED, NM, and BB contributed to patient recruitment and data collection. All authors drafted the manuscript, with support from OPEN Health Communications (funded by Shield). All authors read, revised, and approved the final manuscript for submission.

Supported by: This trial was sponsored by Shield Therapeutics (United Kingdom) Ltd.

Conflicts of interest: Stefanie Howaldt has served as a speaker or has received research or education funding or advisory fees from MSD, AbbVie, Biogen, Celltrion, Hospira, Takeda, Pfizer, Pharmacosmos, Janssen, R-Biopharm, Norgine, Sandoz, and Shield Therapeutics. Eugeni Domènech has served as a speaker or has received research or education funding or advisory fees from MSD, AbbVie, Takeda, Kern Pharma, Pfizer, Janssen, Celgene, Adacyte Therapeutics, Otsuka Pharmaceuticals, Ferring, Shire Pharmaceuticals, Tillots, Thermofisher, Grifols, and Gebro. Carsten Schmidt has received consulting fees from AbbVie, Biogen, Ewopharma, Janssen-Cilag, MSD, Pfizer and Takeda; has provided speaking and teaching services for AbbVie, Berlin-Chemie, Biogen, Ewopharma, Falk, Janssen-Cilag, Merckle, MSD, Norgine, Novartis, Olympus, Pentax, Pfizer, Shire, Shield Therapeutics, Takeda and UCB; and has received grants and research funding from AbbVie. Bernd Bokemeyer has received consulting fees from AbbVie, MSD, Shire, Ferring, UCB, Hospira, Takeda, Movetis, Shield Therapeutics, Pfizer, Biogen, Janssen, Hexal, Celgene, Boehringer, Allergan, and Celltrion; has provided speaking and teaching services for AbbVie, Ferring, MSD, Merckle, Falk, HLR, UCB, Shield Therapeutics, Pfizer, Celltrion, Takeda, Janssen, Mundipharma, and Tillotts; and has received grants and research funding from AbbVie, Ferring, UCB, Given Imaging, Janssen, Takeda, and Pfizer.
