# Corrigendum to: Long-Term Effectiveness of Oral Ferric Maltol vs Intravenous Ferric Carboxymaltose for the Treatment of Iron-Deficiency Anemia in Patients With Inflammatory Bowel Disease: A Randomized Controlled Noninferiority Trial

**DOI:** 10.1093/ibd/izab224

**Published:** 2021-10-06

**Authors:** Stefanie Howaldt, Eugeni Domènech, Nicholas Martinez, Carsten Schmidt, Bernd Bokemeyer

**Affiliations:** 1 Research Institute for IBD–HaFCED e.K., Hamburg, Germany; 2 Gastroenterology and Hepatology Department, Hospital Universitari Germans Trias i Pujol, Badalona, Catalonia, and Centro de Investigación Biomédica en Red en Enfermedades Hepáticas y Digestivas, Madrid, Spain; 3 Gastroenterology Research of America, San Antonio, Texas, USA; 4 Department of Gastroenterology, Hepatology, Endocrinology, Diabetology, and Infectious Diseases, Klinikum Fulda, Fulda, Germany; 5 Gastroenterology Practice Minden and University Hospital Schleswig-Holstein, Campus Kiel, Clinic for Internal Medicine I, Kiel, Germany

In the article titled “Long-Term Effectiveness of Oral Ferric Maltol vs Intravenous Ferric Carboxymaltose for the Treatment of Iron-Deficiency Anemia in Patients With Inflammatory Bowel Disease: A Randomized Controlled Noninferiority Trial”, the legends for Figures 3 and 4 were incorrect.

The incorrect legends read as follows:

“Figure 3. Mean change in Hb over 52 weeks of treatment with oral ferric maltol or IV ferric carboxymaltose (ITT population, observed patients). *P* values shown for test of null hypothesis of inferiority in risk difference with noninferiority margin of 20%.”.

“Figure 4. Patients achieving ≥1 and ≥2 g/dL increases, and normalization of Hb concentration between baseline and week 12 (ITT population with multiple imputation). *P* values shown for test of null hypothesis of inferiority in risk difference with noninferiority margin of 20%.”.

The legends should read as follows:

“Figure 3. Mean change in Hb over 52 weeks of treatment with oral ferric maltol or IV ferric carboxymaltose (ITT population). *P* values shown for least-squares mean change from baseline, difference between groups.”.

“Figure 4. Patients achieving ≥1 and ≥2 g/dL increases, and normalization of Hb concentration between baseline and week 12 (ITT population with multiple imputation). *P* values shown for risk difference between groups.”.

These errors have been corrected online and in print.

