# Supplementary material for: Long-Term Effectiveness of Oral Ferric Maltol vs Intravenous Ferric Carboxymaltose for the Treatment of Iron-Deficiency Anemia in Patients With Inflammatory Bowel Disease: A Randomized Controlled Noninferiority Trial
Source: Inflamm Bowel Dis. 2021 May 14;28(3):373–84. doi: 10.1093/ibd/izab073 (PMC8889281; doi:10.1093/ibd/izab073)

**Supplementary Data Content**

**Major protocol deviations**

According to the trial protocol, major protocol deviations could include the following:

- Patients who received at least 1 dose of a study drug but who did not satisfy major inclusion/exclusion criteria
- Patients who met conditions for withdrawal but who were not withdrawn
- Patients who received a prohibited concomitant medication
- Patients for whom at least 1 major required protocol procedure was not performed (e.g., missing Hb results at baseline or week 12)
- Patients whose Hb value was measured more than 2 weeks (± 14 days) outside the planned Week 12 assessment
- Patients who received the opposite study drug treatment than the one to which they were randomized
- Repeated non-compliance in self-administration of ferric maltol (i.e., <80% or >120% of expected administration of study drug). Where data were missing, the compliance was assessed based on individual visit pill returns; where data existed, the compliance was based on the average compliance in the first 12 weeks of study
- IV iron dosing that did not follow the specific country dosing regimens (i.e. dosing up to week 12 that was ≥ 2 or ≤ 0.5 times the calculated dose based on weight, Hb, and local Prescribing Information)
- Other relevant deviations judged on an individual basis

TABLE S1. Major protocol deviations reported up to week 12

| **Patients with protocol deviation, n (%)** | **Ferric maltol (n = 125)** | **IV ferric carboxymaltose (n = 125)** |
| --- | --- | --- |
| Any | 46 (37) | 34 (27) |
| Inclusion/exclusion criteria | 6 (5) | 7 (6) |
| Study drug administration/dose | 20 (16) | 15 (12) |
| Informed consent | 1 (1) | 0 |
| Visit schedule | 25 (20) | 12 (10) |
| Protocol assessment/procedure | 3 (2) | 3 (2) |
| Other | 1 (1) | 1 (1) |

Patients could have major protocol deviations in more than one category.

Supplementary Figure S1. Mean change in ferritin over 52 weeks of treatment with oral ferric maltol or IV ferric carboxymaltose (intent-to-treat population, observed cases). Abbreviations: ET, end of treatment; IV, intravenous.


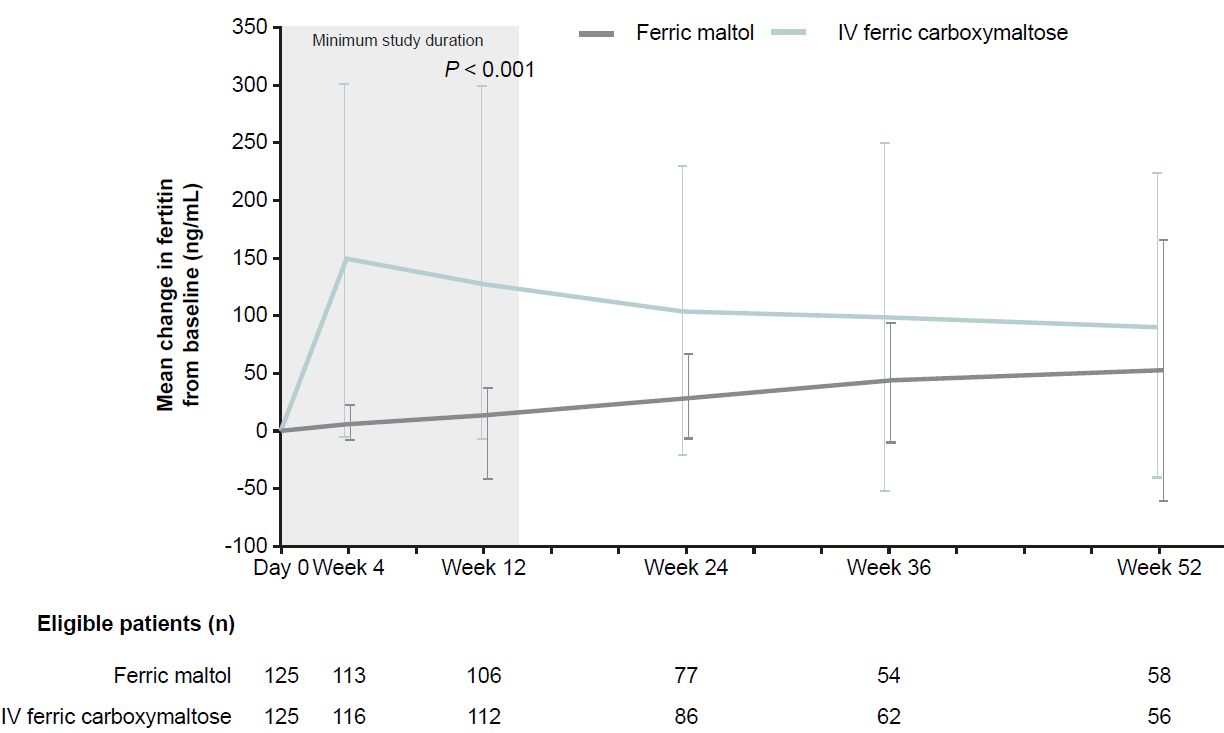


Supplementary Figure S2. Patients achieving normalization of ferritin over time (intent-to-treat population, observed cases). Abbreviation: IV, intravenous.


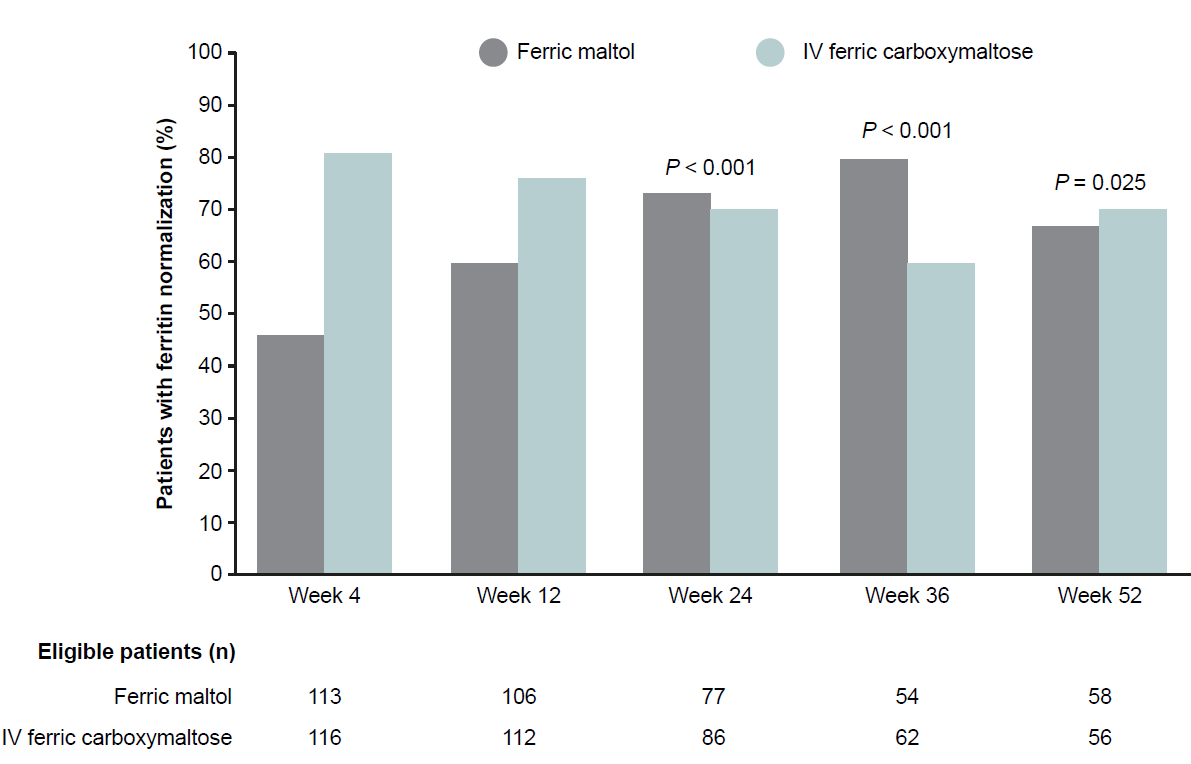

Supplement: izab073_suppl_Supplementary_Material [file izab073_suppl_supplementary_material.docx]
